# Integrative Multi-Omics Analysis Reveals Systemic Transcriptional and Hormonal Reprogramming Associated with a Rice Yellow-Green Leaf Mutant

**DOI:** 10.3390/cimb48080848

**Published:** 2026-08-20

**Authors:** Guang Li, Jiawei Liu, Xiao Yang, Mangu Hu, Yongxiang Huang

**Affiliations:** 1 College of Coastal Agricultural Sciences, Guangdong Ocean University, Zhanjiang 524088, China; liguang12@stu.gdou.edu.cn (G.L.);; 2 College of Agriculture, South China Agricultural University, Guangzhou 510642, China

**Keywords:** *Oryza sativa*, chlorophyll homeostasis, multi-omics analysis, retrograde signaling, hormone reprogramming

## Abstract

Leaf-color mutants are crucial for elucidating chlorophyll metabolism mechanisms. Here, we identified *yel*, a stably inherited rice mutant with a dwarf and yellow-green leaf phenotype controlled by a single recessive nuclear gene. Using BSA-seq, we mapped the candidate causal gene to *OsMPEC*, encoding a magnesium protoporphyrin IX monomethyl ester cyclase with a G→T substitution. Multi-omics analysis revealed that the functional deficiency of OsMPEC protein—despite unchanged transcript levels—triggers global transcriptional repression of the chlorophyll metabolic network. This defect caused distinct metabolic consequences: impaired synthesis at the early stage (yel1) and toxic metabolite accumulation at the later stage (yel2). Furthermore, metabolic collapse induced systemic reprogramming of hormone signaling, shifting from pro-growth to stress and senescence modes. We propose that the *yel* phenotype is associated with a self-reinforcing inhibitory loop of “passive synthesis inhibition” and “active degradation acceleration.” This study clarifies *OsMPEC*’s role in chlorophyll homeostasis and demonstrates how a single genetic defect drives phenotype- through to network-level cascading effects.

## 1. Introduction

Rice is the cornerstone of global food security [1], and its leaf photosynthetic efficiency critically determines yield [2]. Leaf-color mutants serve as classic models for research. Because of their intuitive phenotype and connection to photosynthesis, they are ideal for dissecting the genetic networks governing chlorophyll metabolism, chloroplast development, and photosynthesis [3]. The biosynthesis of chlorophyll is a highly conserved multi-step pathway [4]. One of these steps, a cyclization reaction, is particularly key. It is catalyzed by the enzyme magnesium–protoporphyrin IX monomethyl ester cyclase, which is encoded by the MPEC gene. This reaction forms the characteristic molecular structure of chlorophyll [5,6]. In *Arabidopsis thaliana*, the loss of the homologous gene *CHL27* leads to severe chlorosis and growth inhibition, which highlights its fundamental function [7]. In recent years, leaf-color phenotypes have become increasingly valued by breeders. Consequently, leaf-color mutations have been identified and studied in a wide range of plants, such as maize [8,9], poplar [10,11], tobacco [12,13,14,15], *Arabidopsis thaliana* [16,17,18,19], rice [20,21,22,23,24], *Rosa multiflora* [25,26], and melon [27]. However, most of this research focuses on describing the terminal leaf-color phenotype and its direct biochemical causes [28,29]. A deeper, unresolved question remains. How does an initial metabolic perturbation, like the partial loss of *OsMPEC* function, trigger cellular adaptive responses that ultimately produce the whole-plant developmental program? More specifically, does a local interruption in chlorophyll biosynthesis cause a global reprogramming of the entire metabolic network, and if so, what is the mechanism? Furthermore, which signaling hubs regulate this metabolic state shift to control downstream growth? Answering these questions is crucial for a systems biology understanding of the integrated regulatory link between metabolic homeostasis, signal transduction, and morphogenesis. This study reports a stably inherited rice mutant, yellow-green leaf (*yel*), created by space mutagenesis. The mutant exhibits both a life-long yellow-green leaf phenotype and a significant dwarf phenotype, providing ideal material for exploring the scientific questions mentioned above. The objectives of this study are to investigate key agronomic traits, photosynthetic pigment content, and photosynthetic parameters; to combine these with BSA-seq technology for preliminary localization of the candidate gene region; and to explore the mechanism of the *yel* leaf-color mutation through transcriptomic and metabolomic approaches. We aim to reveal the systemic metabolic reprogramming map triggered by the *OsMPEC* mutation and to elucidate the cross-level regulatory pathway that inhibits plant growth by interfering with the hormone signaling network.

Importantly, while fine-mapping and functional validation of the candidate gene remain as future directions, the primary contribution of this study lies in providing a comprehensive, multi-layered omics dataset that captures the dynamic transcriptional and metabolic landscape of a rice mutant exhibiting yellow-green leaf phenotype. This systems-level resource offers a valuable hypothesis-generating platform for dissecting the regulatory networks linking chloroplast metabolism, hormone signaling, and plant development.

## 2. Materials and Methods

### 2.1. Plant Materials and Growth Conditions

The rice dwarf and yellow-green leaf mutant, yellow-green leaf (*yel*), was used in this study. It was derived from a space-mutagenized population of the indica restorer line HR15. In November 2011, dry seeds of HR15 were carried aboard the Shenzhou-VIII spacecraft and exposed to space conditions for 398 h at orbital altitudes ranging from 200 km (perigee) to 330 km (apogee). After recovery, the mutagenized seeds were germinated alongside non-mutagenized controls, and M_1_ plants were transplanted as single plants. At maturity, the main panicle of each M_1_ plant was harvested individually, and equal amounts of seeds from all main panicles were bulked to constitute the M_2_ generation. From the M_2_ population, we identified a yellow-green dwarf mutant, which was subsequently self-pollinated for four consecutive generations to obtain a stable homozygous line, designated as *yel*. The mutagenesis frequency was not precisely determined in this initial screening. Trait stability was ensured by four consecutive generations of self-pollination. The wild-type (*WT*) HR15 was used as the control. For genetic analysis, reciprocal crosses were performed between *yel* and conventional rice varieties Nipponbare and 02428, and the F_1_ plants were self-pollinated to obtain the F_2_ segregating population. All plant materials were cultivated in the experimental field of the Institute of Agricultural Biotechnology, Guangdong Ocean University. Seeds were germinated in a greenhouse and transplanted to the field three weeks later. Each material was planted in 8 rows with a row length of 2 m, row spacing of 30 cm and plant spacing of 20 cm under single-plant transplanting mode. Standard agronomic practices were adopted for field management [30].

### 2.2. Investigation of Agronomic Traits

At the maturity stage, ten representative individual plants were randomly selected from both the *yel* mutant and WT populations for agronomic trait measurement. The following traits were evaluated: Plant height was measured from the ground surface to the tip of the tallest panicle (excluding awns) using a graduated ruler, with an accuracy of 0.1 cm. Panicle length was measured from the panicle neck node to the tip of the uppermost grain (excluding awns) on the main panicle of each plant. Number of effective panicles per plant was determined by counting all panicles that bore at least one filled grain at maturity. Total grains per panicle and filled grains per panicle were counted manually after threshing the main panicle; filled grains were identified by their plumpness and dark color, while unfilled grains were light and shriveled. The seed setting rate was calculated as (number of filled grains/total grains) × 100%. 1000-grain weight was measured by randomly counting three subsamples of 1000 filled grains from each plant, weighing them on an electronic balance (accuracy 0.01 g), and taking the average. Grain weight per plant was determined by weighing all filled grains harvested from a single plant after drying to constant moisture content (approximately 13% water content). All measurements were performed with three technical replicates per plant, and the mean values were used for statistical analysis.

### 2.3. Measurement of Photosynthetic Pigment Content

The top fully expanded leaves of *yel* and *WT* plants were collected at five key growth stages: the seedling stage, the early tillering stage, the late tillering stage, the heading stage, and the maturity stage. For each sample, 0.1 g of fresh leaf tissue was precisely weighed. The tissue was then immersed in 10 mL of 95% (*v*/*v*) ethanol. Samples were incubated in the dark until the tissues turned completely white. The absorbance of the supernatant was measured at 665 nm, 649 nm, and 470 nm using a UV-Vis spectrophotometer. The concentrations of chlorophyll a (Chl a), chlorophyll b (Chl b), and carotenoids (Car) were calculated according to the formula by Lichtenthaler (1987) [31]. Total chlorophyll content was the sum of Chl a and Chl b [31].

### 2.4. Measurement of Photosynthetic Gas Exchange Parameters

At the four-leaf stage, the third fully expanded leaf from the top was measured between 9:00 and 11:00 a.m. on a sunny day. All measurements were performed using a portable photosynthesis system (LI-6800XT, LI-COR Biosciences, USA; manufactured in Lincoln, Nebraska). The conditions in the leaf chamber were set as follows: photosynthetically active radiation (PAR) at 1000 μmol·m^−2^·s^−1^, CO_2_ concentration at 400 μmol·mol^−1^, and leaf temperature at 28 °C. The net photosynthetic rate (P_n_), stomatal conductance (G_s_), transpiration rate (T_r_), and intercellular CO_2_ concentration (C_i_) were recorded.

### 2.5. BSA-Seq Analysis and Gene Mapping

#### 2.5.1. Population Construction and DNA Bulk Preparation

Thirty individual plants with extreme yellow-green leaf phenotype and thirty plants with normal green leaf phenotype were selected from the F_2_ segregating population derived from the cross of *yel* × Nipponbare. The total F_2_ population comprised 611 individuals (Table 1), which guaranteed sufficient phenotypic segregation accuracy for bulked segregant analysis. Genomic DNA was extracted from each plant via the CTAB method. After precise quantification and purity inspection, equal amounts of genomic DNA from each individual were thoroughly mixed to construct the yellow-green leaf pool (Y-pool) and normal green leaf pool (G-pool) [32]. Genomic DNAs harvested from the two parental lines (*yel* mutant and wild-type Nipponbare) were separately extracted and preserved as genomic reference controls for subsequent variant comparison.

#### 2.5.2. Library Construction, Sequencing, and Data Preprocessing

Library construction and sequencing were commissioned to Biomarker Technologies Co., Ltd. (Beijing, China). Qualified genomic DNA was randomly fragmented by sonication to construct paired-end sequencing libraries with an average insert size of approximately 350 bp. All constructed libraries were subjected to 150 bp paired-end sequencing on an Illumina NovaSeq 6000 platform (Illumina, San Diego, CA, USA). For raw sequencing data preprocessing, low-quality reads, adapter-contaminated reads and reads containing excessive ambiguous bases were eliminated using fastp (v0.23.2) to acquire high-quality clean reads. Clean reads were aligned to the rice reference genome (MSU RGAP 7.0) using BWA-MEM2 (v2.2.1). SAMtools (v1.15) was adopted to sort aligned bam files, remove PCR duplicates and conduct base quality recalibration to ensure reliable variant detection.

#### 2.5.3. Variant Detection and Association Analysis

SNPs and InDels were detected using the Haplotype Caller module of GATK (v4.2.6.1), followed by strict hard filtering. The resulting high-quality variants were then functionally annotated using SnpEff (v5.1), which categorized variants into intergenic regions, introns, regulatory regions and coding sequences; coding variants were further classified into synonymous and non-synonymous substitutions.

Association analysis was performed using two algorithms based on the allele frequencies of the two bulks: the Euclidean distance (ED) method and the Δ(SNP-index) method [33]. The threshold for the ED analysis was set as the median of the genome-wide ED values plus three standard deviations. For the Δ(SNP-index) analysis, a 99% confidence interval was used as the threshold. The final candidate region was defined as the overlapping interval identified by both algorithms to minimize false-positive signals. Subsequent fine mapping narrowed down the candidate locus. Genome-wide variant comparison between parents verified that only *OsMPEC* harbored the causative non-synonymous mutation, and no other functional mutations related to chloroplast or chlorophyll development were detected across the rice genome, which ruled out the involvement of other gene mutations in the leaf chlorosis phenotype and downstream biochemical cascades.

### 2.6. Transcriptome Analysis

#### 2.6.1. RNA Extraction and Library Construction

Leaf samples were collected from both *yel* and WT plants at the seedling stage (specifically, the two-leaf and three-leaf stages). Total RNA was extracted using the RNAprep Pure Plant Kit (Tiangen, Beijing, China). RNA integrity was assessed, and only samples with a RIN (RNA Integrity Number) value greater than 7.0 were used for library construction. Subsequently, mRNA was enriched from the total RNA using Oligo(dT) magnetic beads. Strand-specific sequencing libraries were then constructed and sequenced on an Illumina NovaSeq 6000 platform.

#### 2.6.2. Differential Gene Expression Analysis

The raw sequencing reads were subjected to quality control using fastp. High-quality RNA-seq reads were then aligned to the reference genome using HISAT2 (v2.2.1). String Tie (v2.2.1) was used to assemble transcripts and calculate gene expression levels, quantified as FPKM (Fragments Per Kilobase of transcript per Million mapped reads). Differentially expressed genes (DEGs) were identified using the DESeq2 package (v1.38.3) in R. The specific criteria used for screening DEGs are described in Section 2.8.

### 2.7. Untargeted Metabolomics Analysis

#### 2.7.1. Sample Preparation and LC-MS/MS Analysis

Leaf samples from the seedling stage, belonging to the same batch as those used for RNA-seq, were used for metabolite extraction. The analysis was performed on a Waters ACQUITY UPLC I-Class PLUS system coupled to a Waters Xevo G2-XS QToF high-resolution mass spectrometer (Waters Corporation, Milford, MA, USA). Chromatographic separation was achieved using a Waters ACQUITY UPLC HSS T3 column (1.8 μm, 2.1 × 100 mm). Data were acquired in both positive and negative ion modes.

#### 2.7.2. Data Processing and Screening for Differential Metabolites

The raw mass spectrometry data were preprocessed using Progenesis QI (v3.0). Metabolites were then identified by searching against the public METLIN database and the proprietary Biomarker Technologies database. Subsequently, Principal Component Analysis (PCA) and Orthogonal Partial Least Squares Discriminant Analysis (OPLS-DA) were performed using SIMCA-P (v14.1). These analyses were used to screen for differentially accumulated metabolites (DAMs).

### 2.8. Statistical Analysis and Data Visualization

All physiological and biochemical experiments were performed with three independent biological replicates, each representing an individual plant grown in separate pots under identical conditions. For transcriptomic and metabolomic analyses, RNA-seq and LC-MS/MS measurements were conducted on three individual biological replicates per genotype (WT and *yel*) per developmental stage (two-leaf and three-leaf), with each replicate consisting of leaf tissues pooled from three individual plants to minimize individual variation. Data are presented as the mean ± standard deviation (SD). Student’s *t*-test was used to analyze agronomic and physiological data in SPSS 27. Differences were considered significant at *p* < 0.05 and highly significant at *p* < 0.01. The screening criteria for differentially expressed genes (DEGs) were a false discovery rate (FDR) < 0.01 and an absolute log_2_(Fold Change) ≥ 2. FDR was calculated via the Benjamini–Hochberg method to correct for multiple testing. Differentially accumulated metabolites (DAMs) were screened based on a VIP (Variable Importance in Projection) value > 1.0 derived from the OPLS-DA model, a *p*-value < 0.05 obtained from Student’s *t*-test, and a fold change >1.5 or <0.67. No separate FDR correction was applied to metabolite *p* values; the combined constraints of multivariate VIP score, univariate *p* value and fold change were adopted together to alleviate false positives induced by multiple testing. Gene Ontology (GO) and Kyoto Encyclopedia of Genes and Genomes (KEGG) enrichment analyses were conducted using the clusterProfiler package (v4.6.2) in R. A *p*-value < 0.05 was set as the threshold for significant enrichment. All charts and figures were generated using Origin 2026 and the ggplot2 package in R (v4.5.1). The raw sequencing data have been deposited in the Zenodo repository under the record number 21819416 (https://zenodo.org/records/21819416, accessed on 19 August 2026). The dataset is currently under a temporary private access period for peer review and will be made publicly available upon publication.

## 3. Results

### 3.1. The yel Mutant Exhibits Stable Chlorosis and Dwarf Phenotypes

**Figure 1:**
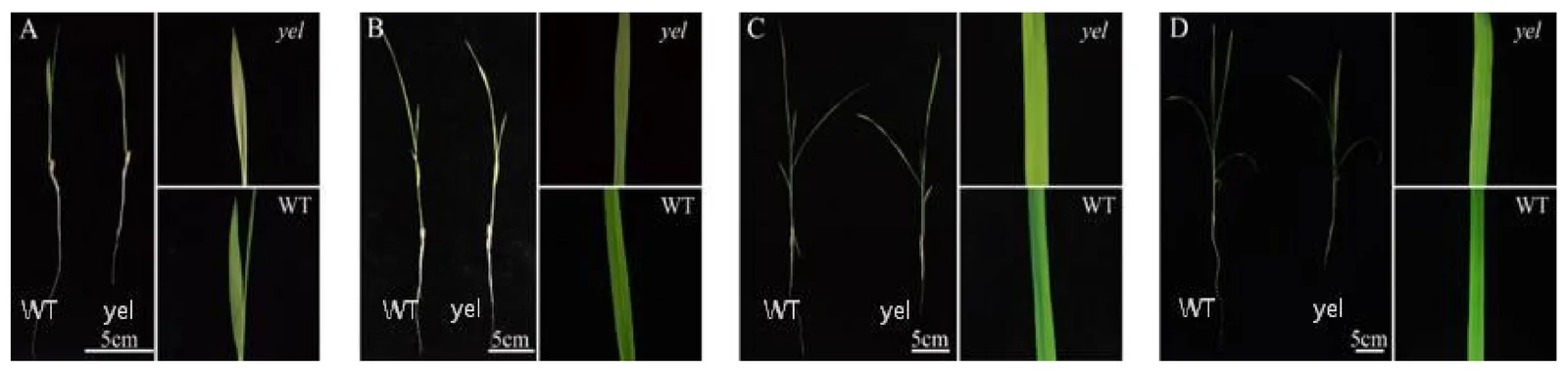
Morphological comparison of the yellow-green leaf mutant and wild-type rice at different stages. (**A**) Two-leaf stage; (**B**) Three-leaf stage; (**C**) Four-leaf stage; (**D**) Five-leaf stage. Plants labeled as “WT” and “*yel*” indicate the respective genotypes in each panel. Plant height data (mean ± SD) for each stage are presented in Section 3.1.

Phenotypic identification revealed significant differences in leaf color and plant architecture between the yel mutant and the wild type. The leaves of yel consistently displayed a yellow-green color throughout their life cycle, a phenotype most pronounced from the seedling to early tillering stage (Figure 1). By the late tillering stage and beyond, although the mutant leaf color showed some recovery, its color value remained significantly lower than that of the wild type, maintaining a distinguishable chlorotic feature. Concurrently, the growth of yel plants was markedly inhibited, with plant height consistently lower than that of the wild-type at all observed stages (Figure 1). Quantitative measurements revealed that at the 2-leaf, 3-leaf, 4-leaf, and 5-leaf stages, the plant height of WT was 10.4 ± 0.7 cm, 15.3 ± 0.9 cm, 19.1 ± 1.1 cm, and 21.2 ± 1.2 cm, respectively, whereas that of yel was 9.8 ± 0.6 cm, 14.4 ± 0.8 cm, 18.0 ± 1.0 cm, and 19.8 ± 1.1 cm, respectively (mean ± SD, *n* = 10). The differences were statistically significant at all four stages (Student’s *t*-test, *p* < 0.05), confirming that the dwarf phenotype is a consistent and measurable trait of the yel mutant throughout early vegetative development.

### 3.2. Key Agronomic Traits of the yel Mutant Are Significantly Affected

**Figure 2:**
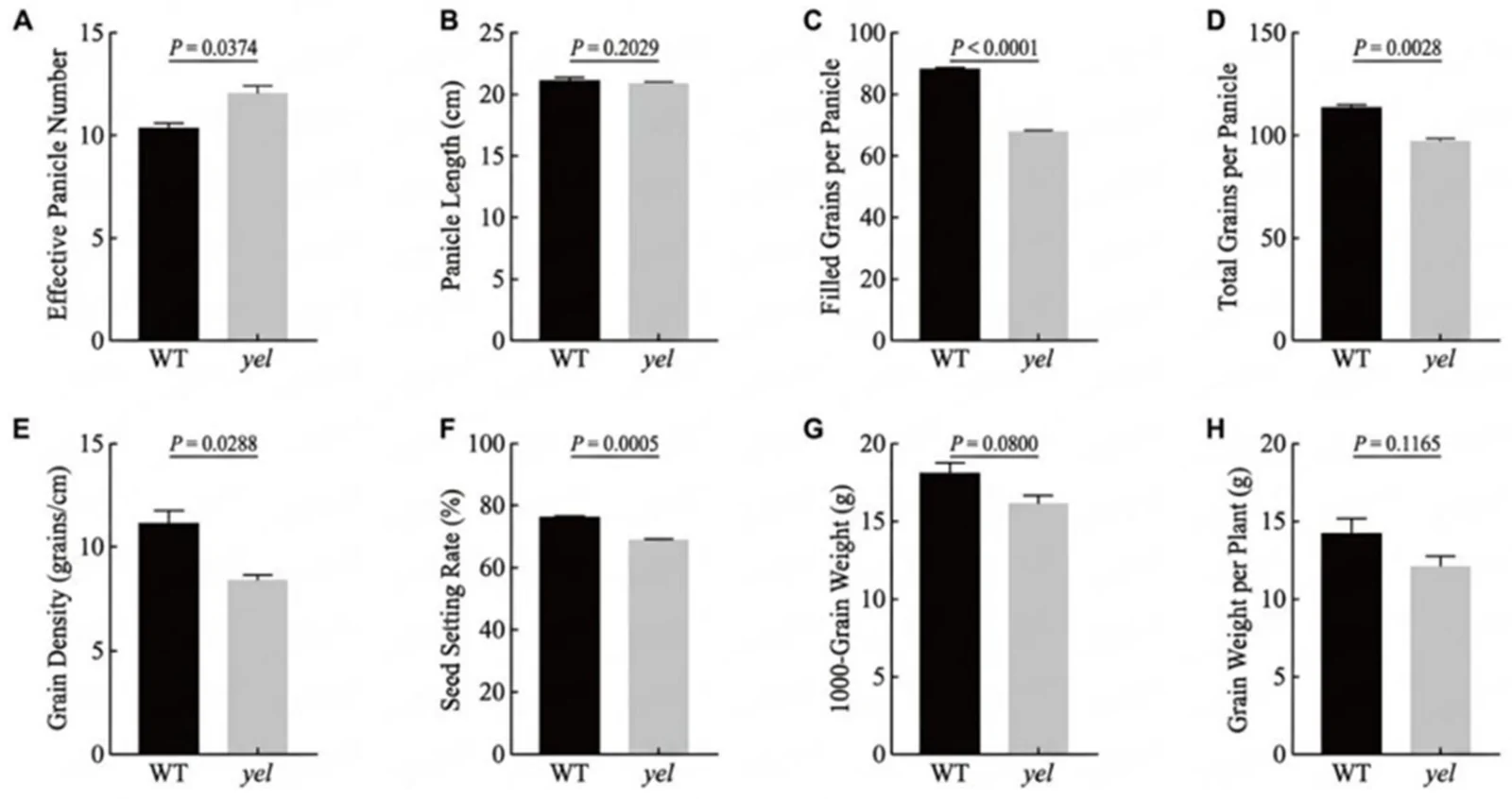
Comparison of yield traits between the yellow-green leaf mutant and wild-type. (**A**) Effective panicles; (**B**) Panicle length; (**C**) Filled grains per panicle; (**D**) Total grains per panicle; (**E**) Grain density; (**F**) Seed setting rate; (**G**) 1000-grain weight; (**H**) Grain weight per plant.

Further comparison of the main agronomic traits between the *yel* mutant and the wild type (WT) revealed that *yel* exhibits unique yield structure characteristics (Figure 2). The mutant showed an advantage in tillering capacity, with a significantly higher number of effective panicles per plant compared to the wild type. However, several key aspects of its reproductive development were significantly suppressed. Analysis of panicle traits showed that the total grains per panicle, filled grains per panicle, and grain density per panicle were all significantly reduced in the mutant, with a highly significant decrease in seed setting rate. Although the panicle length, 1000-grain weight, and grain weight per plant of the mutant showed a decreasing trend, the differences were not significant. Taken together, these results demonstrate a complex yield structure phenotype in the *yel* mutant, characterized by an increased tiller number but significant defects in panicle development and seed setting.

### 3.3. Photosynthetic Pigment Synthesis Is Severely Impaired in the yel Mutant

**Figure 3:**
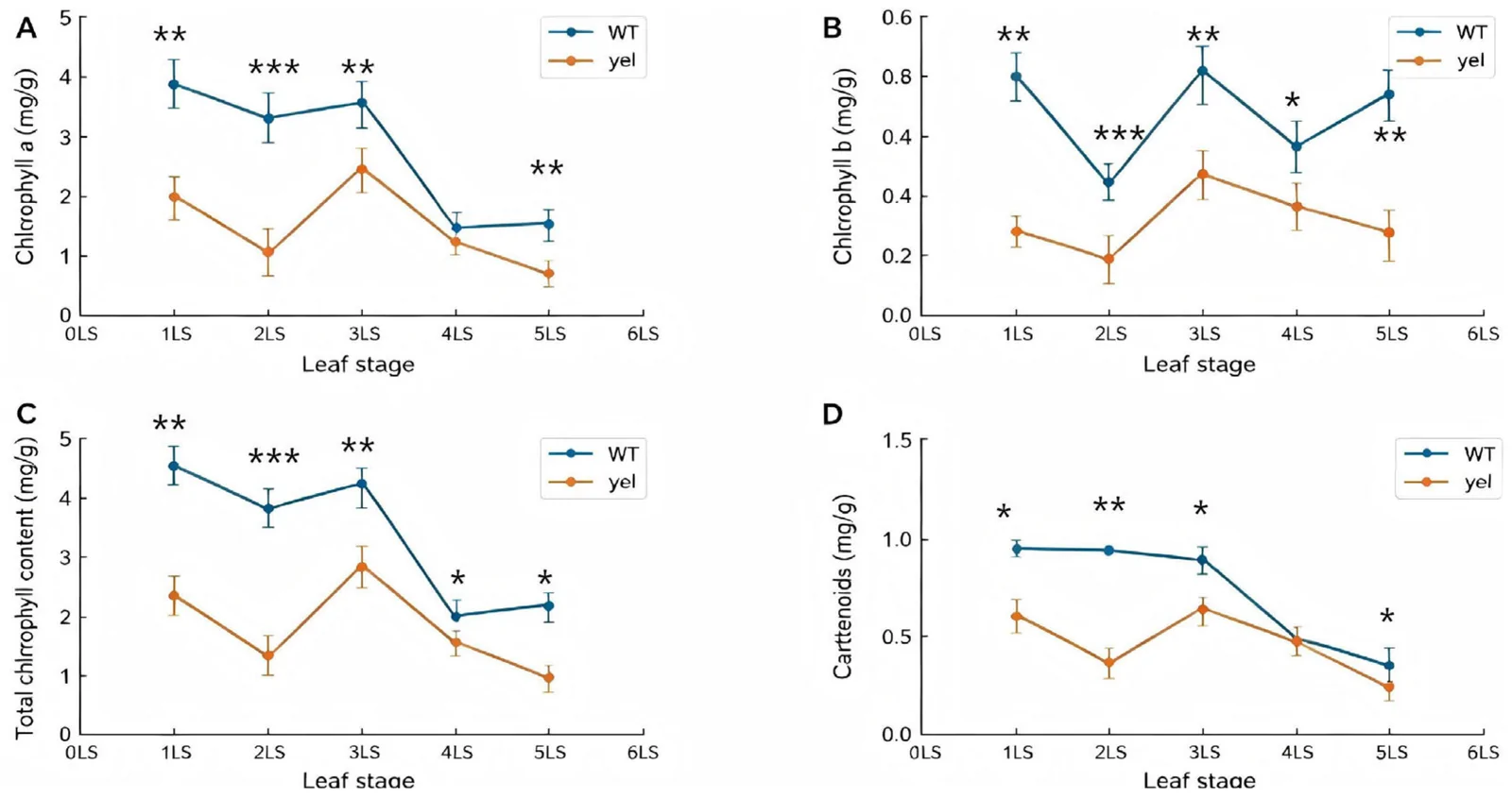
Comparison of photosynthetic pigment content between the yellow-green leaf mutant and wild-type. (**A**) Chlorophyll a; (**B**) Chlorophyll b; (**C**) Total chlorophyll; (**D**) Carotenoids. 0LS–5LS represent successive leaf-development stages. Asterisks indicate significant differences between WT and yel at each leaf stage: * *p* < 0.05, ** *p* < 0.01, *** *p* < 0.001; no asterisk means no significant difference.

The content of chlorophyll, a vital photosynthetic pigment in rice chloroplasts, and its composition directly and significantly influence the plants photosynthetic capacity. We measured the leaf chlorophyll content of the *yel* mutant and WT at different growth stages (Figure 3). The results consistently showed that the contents of chlorophyll a, chlorophyll b, and carotenoids were significantly lower in the *yel* mutant than in the WT across all tested stages. The difference was most pronounced at the seedling establishment stage, where the total chlorophyll content of the *yel* mutant was only 33.76% of that in the WT. This severe and persistent reduction in major photosynthetic pigments is consistent with the visible yellow-green leaf phenotype of the *yel* mutant.

### 3.4. Photosynthetic Efficiency Is Significantly Reduced in the yel Mutant.

**Figure 4:**
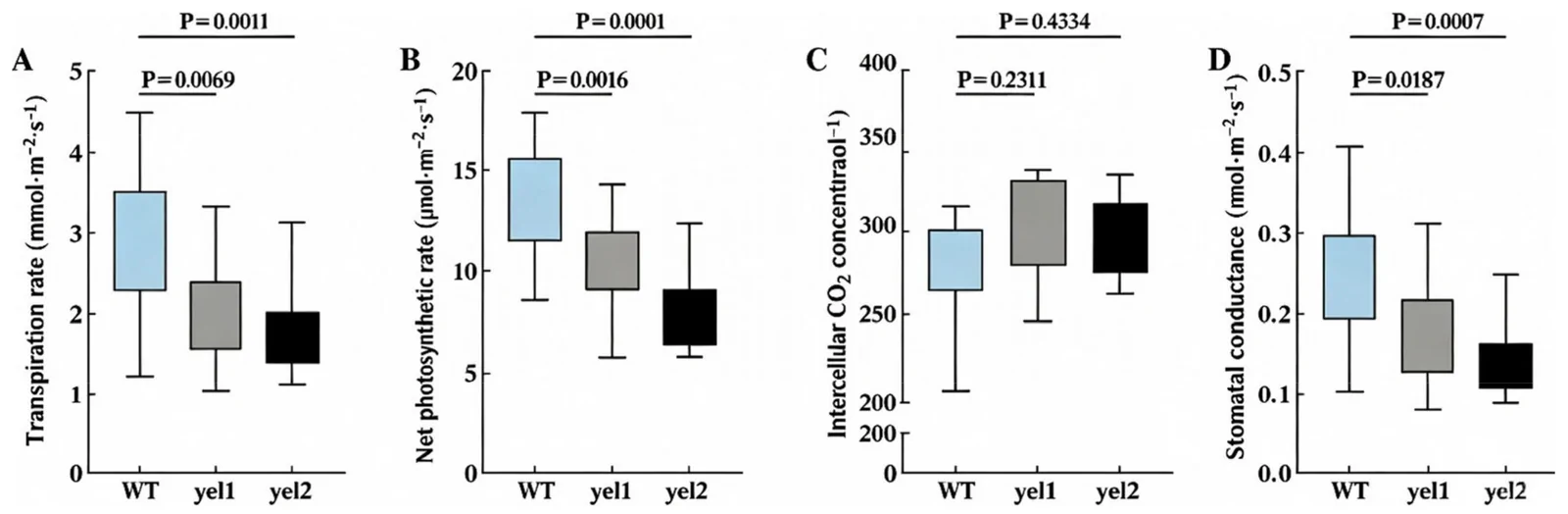
Comparison of photosynthetic parameters between the yellow–green leaf (*yel*) mutant and wild–type (WT) at the seedling stage. (**A**) Transpiration rate (T_r_); (**B**) Net photosynthetic rate (P_n_); (**C**) Intercellular CO_2_ concentration (C_i_); (**D**) Stomatal conductance (G_s_). Blue bars represent WT, gray bars represent *yel* at the two–leaf stage (yel1), and black bars represent *yel* at the three–leaf stage (yel2). Data are shown as mean ± SD (n = 3). Horizontal brackets above the bars indicate the genotype comparisons: lower bracket for WT vs. yel1 and upper bracket for WT vs. yel2, with the corresponding *p*-values shown above each bracket (Student’s *t*-test; *p* < 0.05 was considered statistically significant). All panels share the same color coding and annotation scheme.

To assess the functional impact of the pigment deficiency, we measured photosynthetic gas exchange parameters at the three-leaf stage (Figure 4). The results revealed a significant impairment of photosynthetic efficiency in the *yel* mutant. Compared to the WT, its net photosynthetic rate (Pn), stomatal conductance (Gs), and transpiration rate (Tr) were all highly significantly reduced, by 42.28%, 42.02%, and 37.50%, respectively. In contrast, the intercellular CO_2_ concentration (Ci) was slightly elevated by 10.47%, although this difference was not statistically significant. This finding suggests that the reduced photosynthetic rate in *yel* is likely due to non-stomatal limitations rather than a reduced CO_2_ supply. Changes in plant physiological characteristics or drastic shifts in environmental conditions can affect photosynthesis. In this study, the significant reduction in all photosynthetic pigment contents in the *yel* mutant is the likely cause for its decreased photosynthetic efficiency and the observed inhibition of photosynthesis.

### 3.5. The yel Mutant Trait Is Controlled by a Single Recessive Nuclear Gene

Observations over three generations of self-pollination confirmed that the mutant traits of the *yel* mutant are stably inherited. At the seedling stage, phenotypes of progeny from reciprocal crosses between the *yel* mutant and two conventional rice varieties, Nipponbare and 02428, were investigated. The F_1_ generation all displayed normal green leaf color. In the F_2_ population, clear segregation occurred, with phenotypes resembling both parents emerging. The plants could thus be distinguished into two groups with distinct leaf colors: normal green seedlings and yellow-green chlorotic seedlings (Table 1). A chi-square test showed that the ratio of normal to mutant plants was approximately 3:1, fitting the expected segregation ratio. This indicates that the mutant phenotype is controlled by a single recessive nuclear gene. Combined with preceding quantitative assessments of photosynthetic pigments and yield-related agronomic traits (Figure 2 and Figure 3), we verified that this single recessive mutation severely disrupts chlorophyll homeostasis and photosynthetic performance, leading to suppressed plant growth and declined yield. These quantitative results establish a solid biochemical link between this monogenic recessive trait and perturbed chlorophyll metabolism.

### 3.6. BSA-Seq Identifies a Candidate Locus on Chromosome 5 Associated with the yel Mutant Trait

#### Sequencing Quality and Variant Detection

Sequencing of the two parental lines (FY and QB) and the two bulked pools (H1-G and Yellow) generated high-quality clean data, with Q30 values exceeding 92% and average sequencing depths ranging from 31× to 38× across all samples. The mapping rates against the reference genome were consistently above 97%, and genome coverage (1×, 5×, and 10×) was satisfactory for subsequent analysis (Supplementary Tables S1 and S2). Based on these reliable data, association analysis was conducted using both SNP-index and Euclidean distance (ED) algorithms. Preliminary analysis of SNPs and InDels identified multiple candidate regions on chromosomes 2 and 5 (Supplementary Tables S3 and S4). By intersecting the candidate intervals from both types of molecular markers, we ultimately narrowed down the candidate regions to four intervals totaling 4.34 Mb (Table 2). Most critically, a major locus spanning 3.54 Mb (17,836,161–21,378,705 bp) on chromosome 5 was identified as the primary candidate locus. Although two other minor associated intervals, totaling only 0.04 Mb, were also detected on chromosome 5, they were disregarded in subsequent analyses due to their small size, sparse gene content, and the absence of any known chlorophyll metabolism-related genes.

### 3.7. Candidate Gene Identification and Functional Annotation

**Figure 5:**
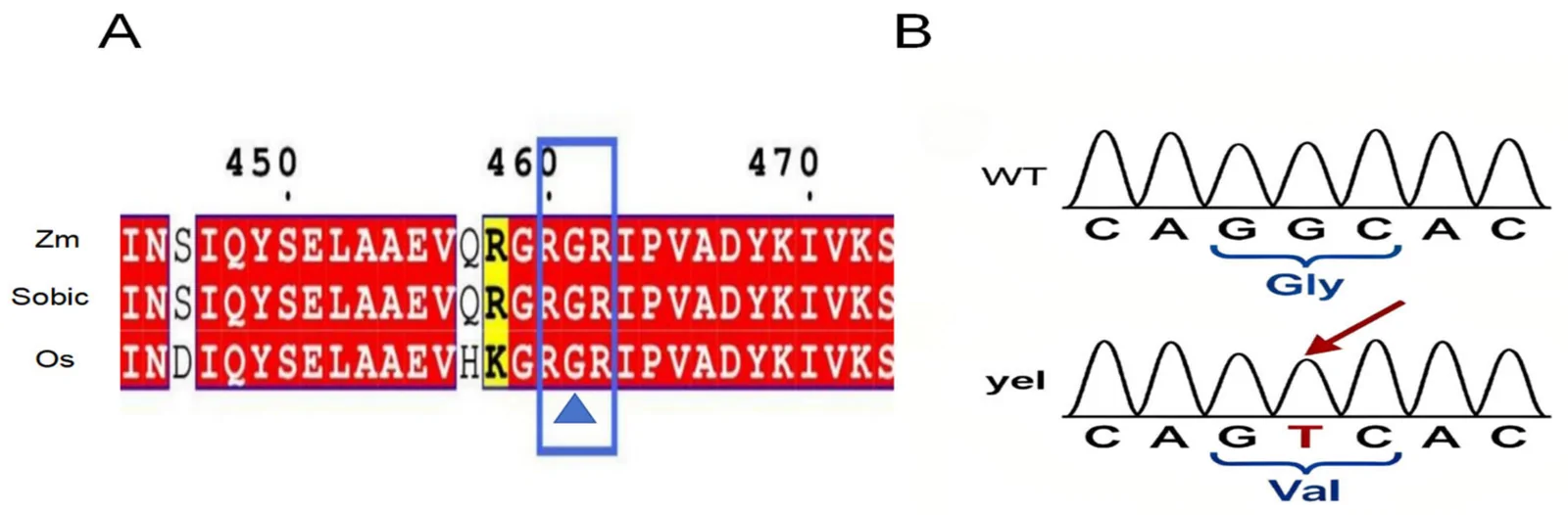
Evolutionary conservation analysis of the mutated residue and schematic validation of the causative point mutation in *OsMPEC* (**A**) Multiple sequence alignment of MPEC homologous proteins from maize (*Zea mays*, Zm), sorghum (*Sorghum bicolor*, Sobic), and rice (*Oryza sativa*, Os). The conserved glycine residue at position 461 is enclosed within the blue rectangular box, which exhibits absolute conservation across the three gramineous species. (**B**) Schematic illustration of Sanger sequencing chromatograms showing the G-to-T single-nucleotide transversion. The wild-type carries codon GGC encoding glycine (Gly); the *yel* mutant bears a G > T substitution altering the codon to GTC, which translates to valine (Val) and results in the Gly461Val missense mutation.

A total of 640 protein-coding genes were annotated within the 4.34 Mb candidate region. Functional annotation and GO enrichment analysis indicated numerous genes associated with chloroplast physiology and primary metabolism (Supplementary Tables S5 and S6). It is necessary to clarify that all candidate genes listed in Table 3 are annotated against the Oryza sativa subsp. japonica Nipponbare reference genome. Os05g0560300 (designated as OsMPEC in our study) is the candidate locus identified in the indica restorer line HR15 background used in this experiment; this gene was not included among the two genes obtained from KEGG pathway enrichment and thus does not appear separately in Table 3. OsMPEC encodes magnesium–protoporphyrin IX monomethyl ester cyclase (MgPEC), a crucial catalytic enzyme indispensable for chlorophyll biosynthesis. More importantly, combined BSA-seq variant screening, Sanger sequencing and homologous sequence alignment confirmed a G→T single-nucleotide substitution within the coding region of OsMPEC in the yel mutant, which leads to a missense amino acid alteration (Figure 5).

More importantly, combined BSA-seq variant screening, Sanger sequencing and homologous sequence alignment confirmed a G→T single-nucleotide substitution within the coding region of *OsMPEC* in the yel mutant, which leads to a missense amino acid alteration (Figure 5). Collectively, KEGG enrichment revealed that the candidate interval harbours multiple genes involved in chlorophyll anabolism, implying overall perturbation of chlorophyll metabolism in this region. Although *OsMPEC* was not captured by pathway-level enrichment statistics, the discovery of the detrimental missense mutation within this functionally critical MgPEC-coding gene verifies that impaired chlorophyll biosynthesis driven by *OsMPEC* dysfunction is the primary cause of leaf chlorosis. The pathway enrichment trend and single-gene mutation evidence corroborate one another.

### 3.8. Transcriptome Analysis Reveals Global Transcriptional Reprogramming in the yel Mutant

#### 3.8.1. A Single-Gene Mutation Triggers a Cascade of Transcriptional Changes

**Figure 6:**
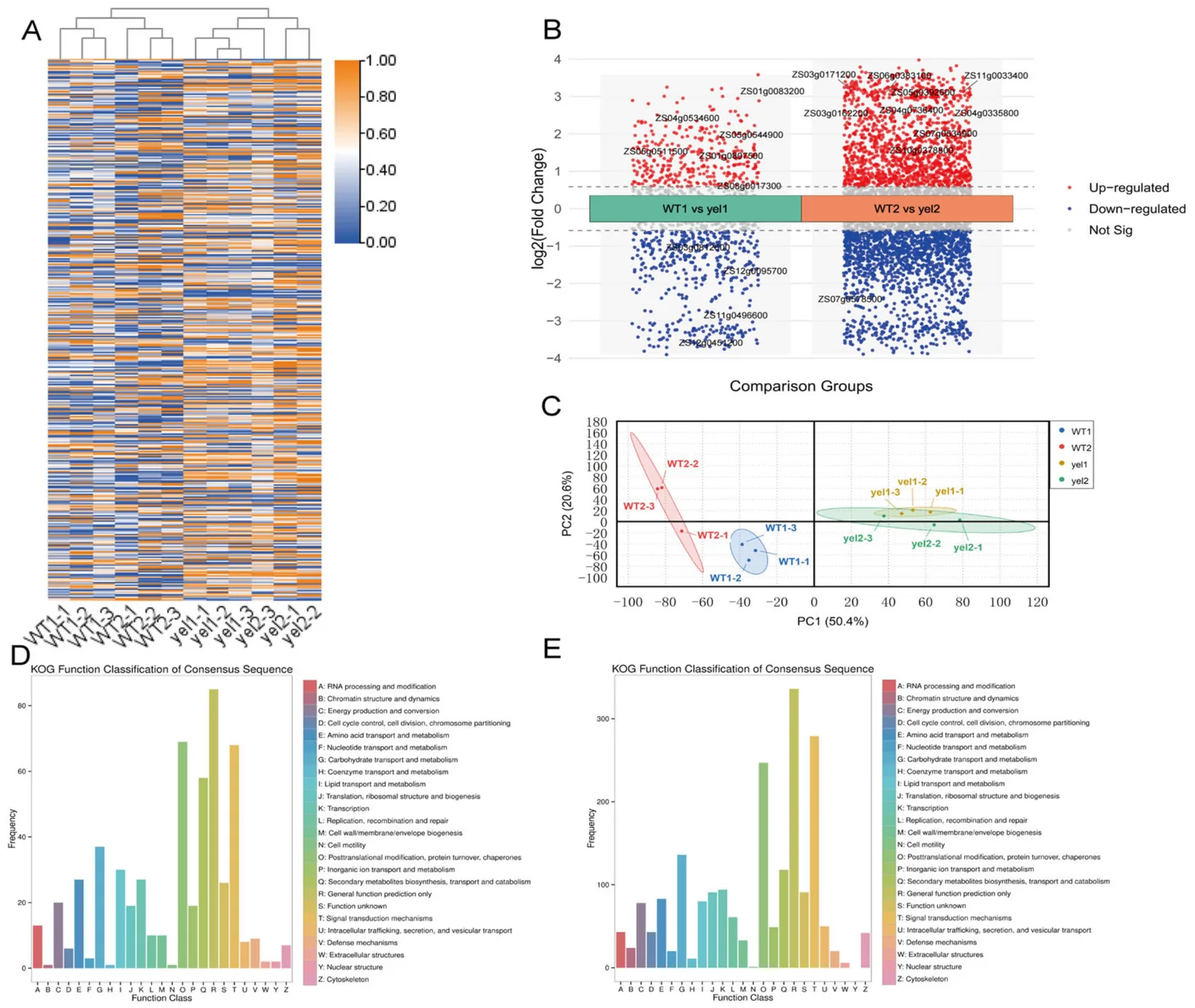
Transcriptome analysis of the yellow-green leaf mutant and wild-type. (**A**) Heatmap of differentially expressed genes. (**B**) Volcano plot of DEGs. (**C**) Principal Component Analysis (PCA). (**D**) KOG functional classification for yel1 vs. WT1. (**E**) KOG functional classification for yel2 vs. WT2.

To dissect the molecular cascade initiated by the *yel* mutation, we performed RNA-seq analysis on leaves at two developmental stages: the two-leaf (yel1) and three-leaf (yel2) stages. The results unequivocally show that the single-gene defect in *OsMPEC* induced a drastic and rapidly amplifying transcriptional reprogramming. Principal component analysis (PCA) revealed a clear separation between mutant and wild-type samples, with the first principal component (PC1) capturing a remarkable 50.4% of the total variance, underscoring the profound and systemic impact of the mutation. The tight clustering of biological replicates within each group confirmed the high reliability of our data (Figure 6C). A heatmap of gene expression visually reinforces this finding, illustrating the starkly different transcriptional landscapes between the two genotypes (Figure 6A). This transcriptional response was not static but evolved dramatically over a short developmental window.

At the two-leaf stage, we identified 1104 differentially expressed genes (DEGs). By the three-leaf stage, this number surged to 4115 (Figure 6B), reflecting dramatic amplification of initial metabolic perturbation. We next conducted KOG functional classification (Figure 6D,E). Despite the largely similar overall KOG category distribution between the two stages, internal compositional differences clearly uncover the two-stage chlorosis progression. Act One (yel1, two-leaf stage): Transcriptional changes were restricted to core information processing (Transcription K, Translation J), accompanied by severe suppression of chloroplast assembly, photosynthesis and energy metabolism, directly stemming from *OsMPEC*-dependent chlorophyll biosynthesis defects. This represents early targeted adjustment of essential cellular and photosynthetic machinery. Act Two (yel2, three-leaf stage): The focused response broadened extensively. Photosynthetic inhibition persisted, while genes for stress response, secondary metabolism, transport and senescence became prevalent; meanwhile, poorly characterized and unknown-function genes increased markedly. This marks the transition from precise adaptive tuning to systemic transcriptional and metabolic disorder.

In brief, the single-gene mutation of *OsMPEC* initiates a progressive genome-wide transcriptional cascade: starting from localized photosynthetic defects and gradually escalating into systemic stress and metabolic chaos.

#### 3.8.2. KEGG Enrichment Analysis: Evolution from Primary Metabolic Disturbance to Systemic Stress

**Figure 7:**
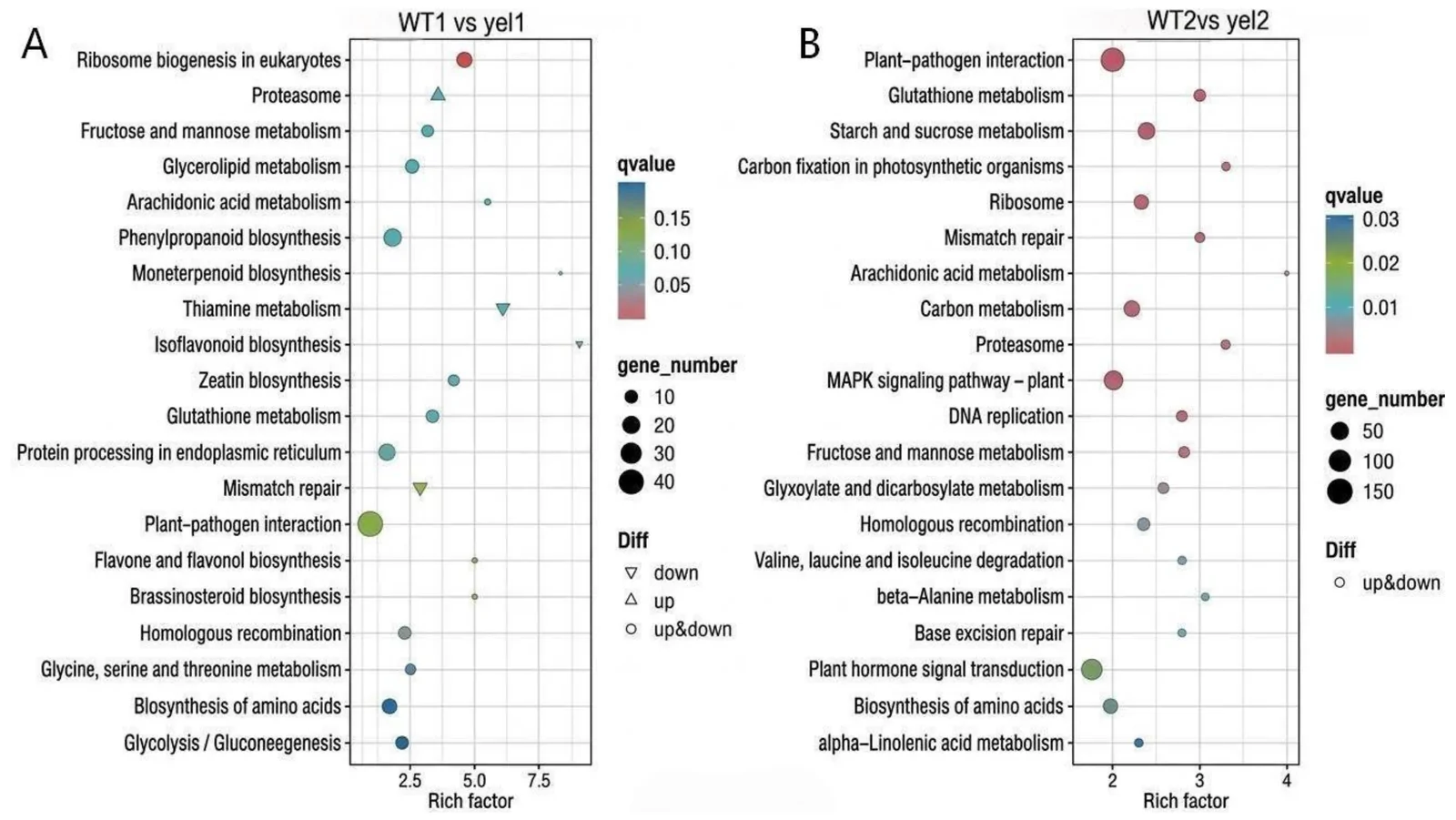
KEGG pathway enrichment analysis of differentially expressed genes (DEGs). (**A**) Bubble chart for the WT1 vs. yel1 comparison. (**B**) Bubble chart for the WT2 vs. yel2 comparison.

KEGG enrichment analysis was performed to identify the key biological pathways associated with the differentially expressed genes (DEGs). The analysis revealed a clear temporal shift in pathway enrichment characteristics from the two-leaf to the three-leaf stage (Figure 7). Although the two bubble plots exhibit similar overall layouts, distinct discrepancies in q-value range, enriched pathway quantity and enrichment strength distinguish the two developmental phases.

At the two-leaf stage (WT1 vs. yel1), transcriptional disturbance was localized and mild. Only a limited number of pathways were enriched, with q-values predominantly concentrated between 0.05–0.15, indicating weak statistical significance. Few pathways reached prominent enrichment intensity; fructose and mannose metabolism was among the handful of pathways approaching the significance threshold (Figure 7A). This demonstrates that the mutation initially triggered slight perturbations confined to discrete primary metabolic pathways. By the three-leaf stage (WT2 vs. yel2), the mutant transcriptome underwent comprehensive global reprogramming. Far more biological pathways were recruited into enrichment, accompanied by drastically reduced q-values (mostly below 0.03) and elevated rich factors. The greatly enhanced statistical confidence and expanded enrichment scope collectively reflect the gradual deterioration from localized mild metabolic disturbance to systemic transcriptional disorder driven by *OsMPEC* dysfunction.

At this stage, DEGs were highly and significantly enriched in numerous key pathways (all q-values ≤ 0.04), which can be categorized into three core functional modules (Figure 7B): Energy and Carbon Metabolism: Pathways including starch and sucrose metabolism, carbon fixation, and carbon metabolism were enriched, pointing to a collapse of the mutant energy production and synthesis capabilities. Stress Response and Defense: The enrichment of pathways such as plant-pathogen interaction, glutathione metabolism, and the MAPK signaling pathway indicates that the cells had initiated broad defense mechanisms to cope with oxidative stress and homeostatic imbalance. Cellular Homeostasis Maintenance: Pathways involving the ribosome, proteasome, and mismatch repair were also enriched, reflecting severe abnormalities in protein synthesis, degradation, and the maintenance of genetic stability.

In summary, the comparison between the two stages reveals a progression from a minor, localized metabolic disturbance to a global, multi-system stress response. This molecular-level shift is highly consistent with the phenotypic peak of leaf chlorosis during the tillering stage, confirming the temporal coherence between phenotype severity and systemic imbalance of the internal regulatory network.

#### 3.8.3. Expression Changes in Key Genes in Chlorophyll Metabolism

Despite the Porphyrin and chlorophyll metabolism pathway not being significantly enriched in the KEGG analysis, a targeted investigation of its key genes was performed, as it contains the candidate causal gene *OsMPEC*. This revealed that chlorophyll homeostasis in the *yel* mutant was negatively impacted in both the synthesis and degradation aspects (Table 4). On the synthesis side, a key rate-limiting step was disrupted. The expression of protochlorophyllide reductase (*OsPORA*), which catalyzes the final light-dependent synthesis step, was significantly suppressed (log_2_FC from −2.03 to −2.48, FDR < 0.01). This downregulation inhibited the primary chlorophyll biosynthesis pathway and directly halted production. Simultaneously, the chlorophyll degradation pathway was systematically activated. Key genes in this multi-step process showed significant upregulation. These included chlorophyllase (*OsCHL*) for the initial step, magnesium chelatase for dechelation, and red chlorophyll catabolite reductase (*OsRCCR*) for later-stage degradation. This coordinated upregulation indicates that the cell initiated an efficient degradation program. In summary, the transcriptional dysregulation in the *yel* mutant followed a clear pattern: the synthesis pathway was suppressed while the degradation pathway was activated. This provides the most direct mechanistic explanation for the plant severe chlorosis phenotype at the molecular level. Most importantly, the analysis pinpointed the direct molecular basis of the chlorosis phenotype. The expression of a key synthesis gene (*OsPORA*) was significantly repressed, while multiple degradation genes were coordinately upregulated. This systemic reprogramming at the transcript level suggested corresponding changes in cellular metabolites. To validate this inference and analyze the mutation functional consequences from a biochemical perspective, we subsequently conducted a non-targeted metabolomics analysis.

### 3.9. Metabolomics Analysis Reveals the Biochemical Phenotype of the yel Mutant

#### 3.9.1. Overview of Differential Metabolites

To elucidate the biochemical consequences of the observed transcriptomic shifts, we conducted non-targeted metabolomics profiling. The analysis revealed a clear metabolic distinction between the wild-type and *yel* mutant samples.

**Figure 8:**
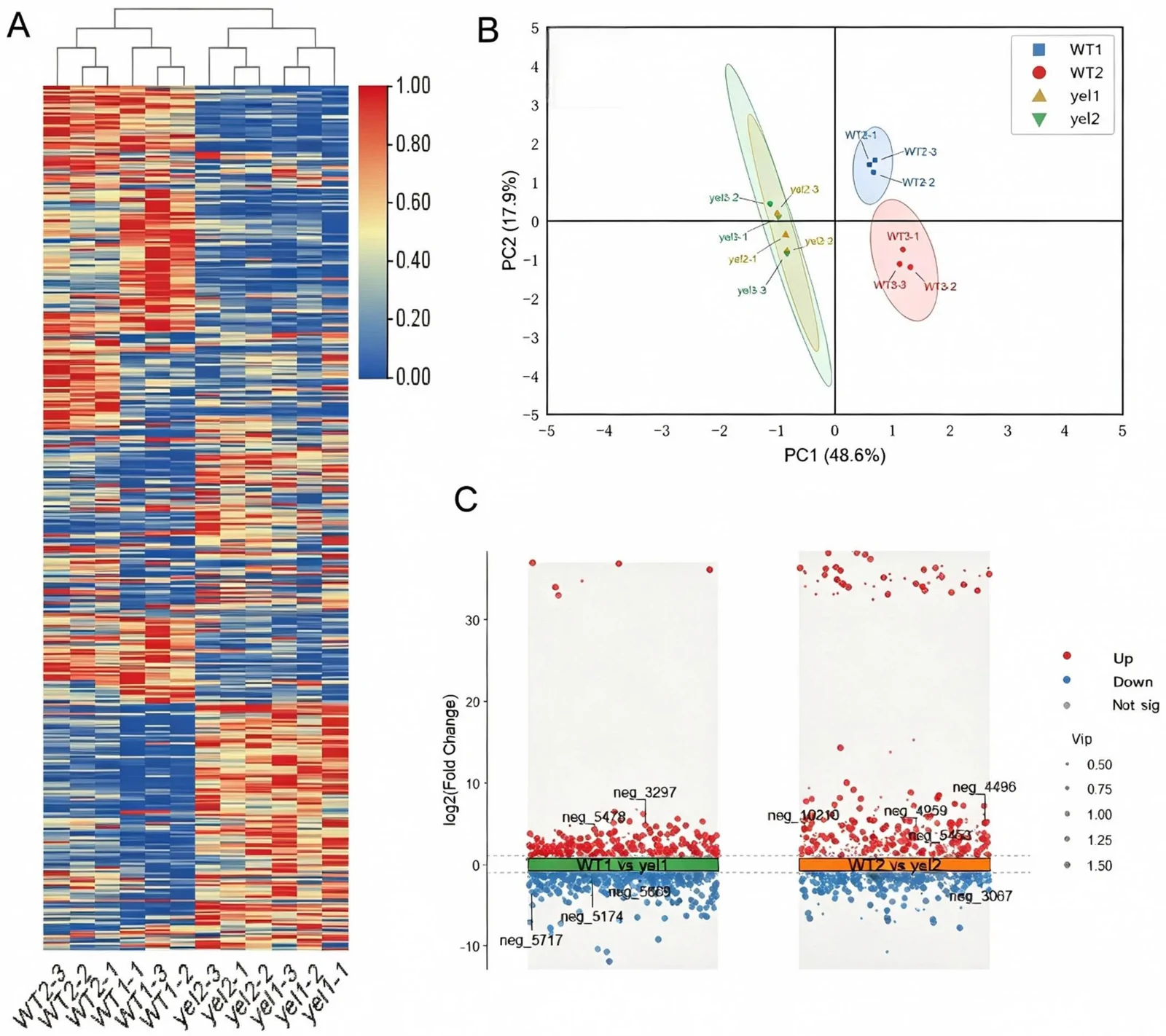
Metabolome analysis of the yellow−green leaf mutant and wild−type. (**A**) Heatmap of differential metabolites. (**B**) PCA of differential metabolites. (**C**) Volcano plot of differential metabolites.

Both heatmap clustering (Figure 8A) and Principal Component Analysis (PCA) (Figure 8B) demonstrated a robust separation of the two genotypes. The PCA results were particularly telling, with the first principal component (PC1) alone accounting for 48.60% of the total inter-group variation, confirming a substantial metabolic reprogramming in the mutant. The *OsMPEC* gene mutation resulted in an extensive and profound remodeling of the metabolic network. Analysis of the volcano plot (Figure 8C) identified 2101 differential metabolites (DMs) in total. At the two-leaf stage (yel1), we identified 1428 DMs (633 upregulated, 795 downregulated). At the three-leaf stage (yel2), the number of DMs reached 1486 (633 upregulated, 853 downregulated).

#### 3.9.2. KEGG Enrichment Analysis: A Cascade from Porphyrin Metabolism Disorder to Systemic Metabolic Dysregulation

**Figure 9:**
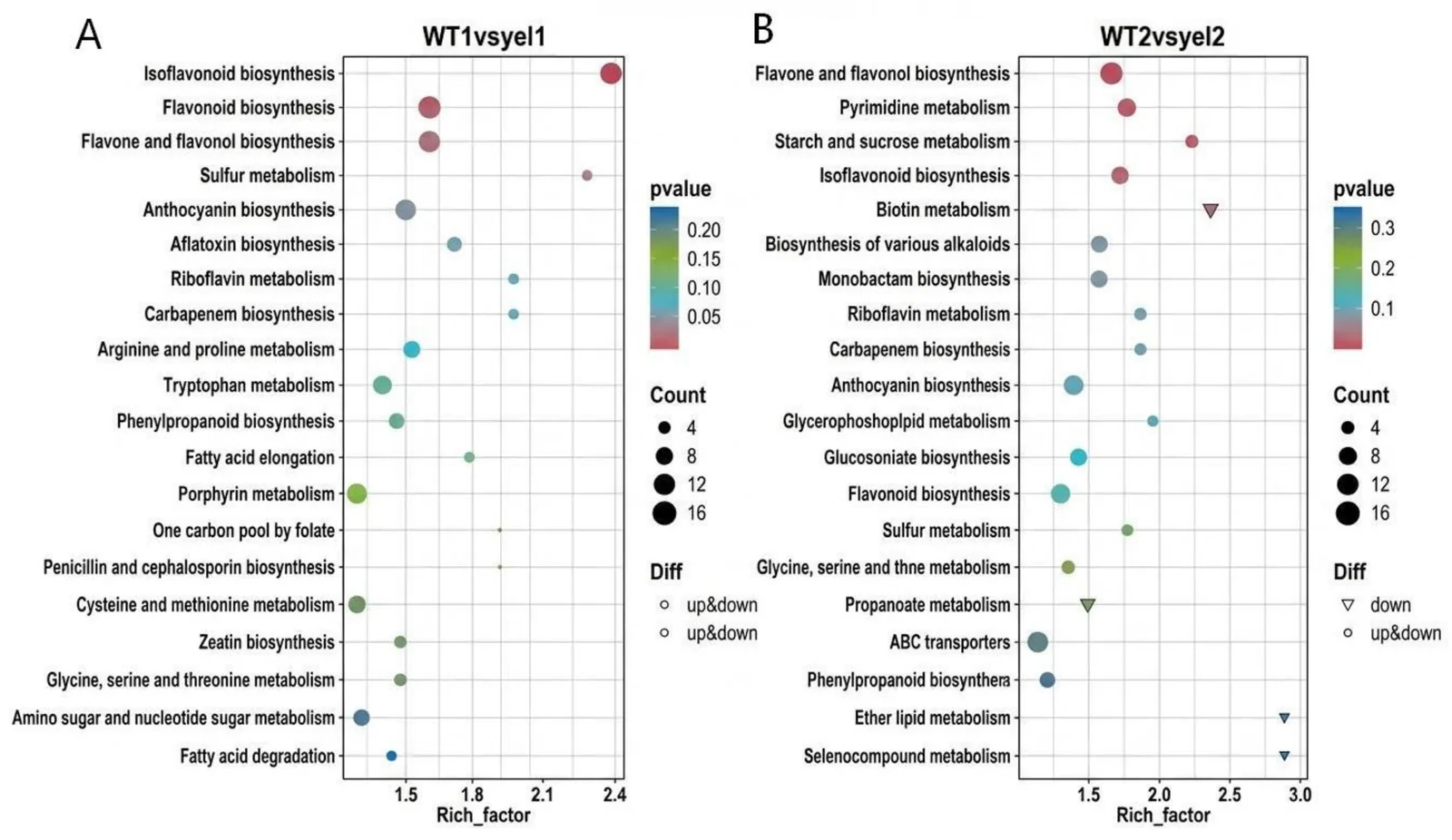
KEGG pathway enrichment analysis of differentially expressed metabolites (DEMs). (**A**) Bubble chart for the WT1 vs. yel1 comparison. (**B**) Bubble chart for the WT2 vs. yel2 comparison.

KEGG pathway enrichment analysis revealed that the *OsMPEC* mutation triggered a cascading metabolic failure, which intensified from the two-leaf to the three-leaf stage (Figure 9). Initially, at the two-leaf stage, the metabolic disruption was targeted. As expected, Porphyrin metabolism was the most significantly enriched pathway, confirming a direct blockage of chlorophyll biosynthesis at the biochemical level (Figure 9A). In response, the plant activated defense mechanisms, evidenced by the significant enrichment of secondary metabolite pathways like Flavone and flavonol biosynthesis. This targeted disruption escalated to systemic dysregulation by the three-leaf stage (Figure 9B). While the initial disorders persisted and worsened, two new critical pathways became significantly affected: Starch and sucrose metabolism and Pyrimidine metabolism. The impairment of starch and sucrose metabolism indicated a failure in primary energy systems, likely due to reduced photosynthetic capacity. The disruption of pyrimidine metabolism pointed to severe impacts on fundamental cellular processes, including nucleic acid synthesis and energy cycling, signifying a widespread collapse of the metabolic network.

In summary, the KEGG analysis accurately delineates a cascading amplification process that originates from the specific dysregulation of a single target (porphyrin metabolism). This, in turn, triggers a defensive stress response, ultimately culminating in the systemic breakdown of core life-sustaining pathways, such as energy and nucleic acid metabolism. This metabolic progression is in strong agreement with the transcriptomic analysis, collectively revealing the causal mechanism underlying the *yel* mutation.

### 3.10. Integrative Analysis: A Causal Chain from Gene Mutation to Systemic Stress

To systematically evaluate the correspondence between transcriptional and metabolic alterations in the *yel* mutant, we mapped the differentially expressed genes and differentially accumulated metabolites onto the chlorophyll biosynthesis and degradation pathways, as well as the plant hormone signaling networks. This integrated analysis allowed us to identify key regulatory nodes where transcriptomic changes correlate with metabolic shifts, providing a systems-level view of the molecular cascades triggered by the MPEC defect. To reveal the fundamental mechanism of chlorosis in the *yel* mutant, we integrated the transcriptome and metabolome data. The analysis established a clear causal chain: the primary *OsMPEC* mutation represses the chlorophyll biosynthesis pathway at the transcriptional level. This specific metabolic block then triggers a systemic cascade, including a plant-wide reprogramming of the hormone signaling network, which ultimately drives the severe chlorotic phenotype and systemic stress.

#### 3.10.1. Integrative Analysis: A Cascade from Transcriptional Repression to Systemic Stress

**Figure 10:**
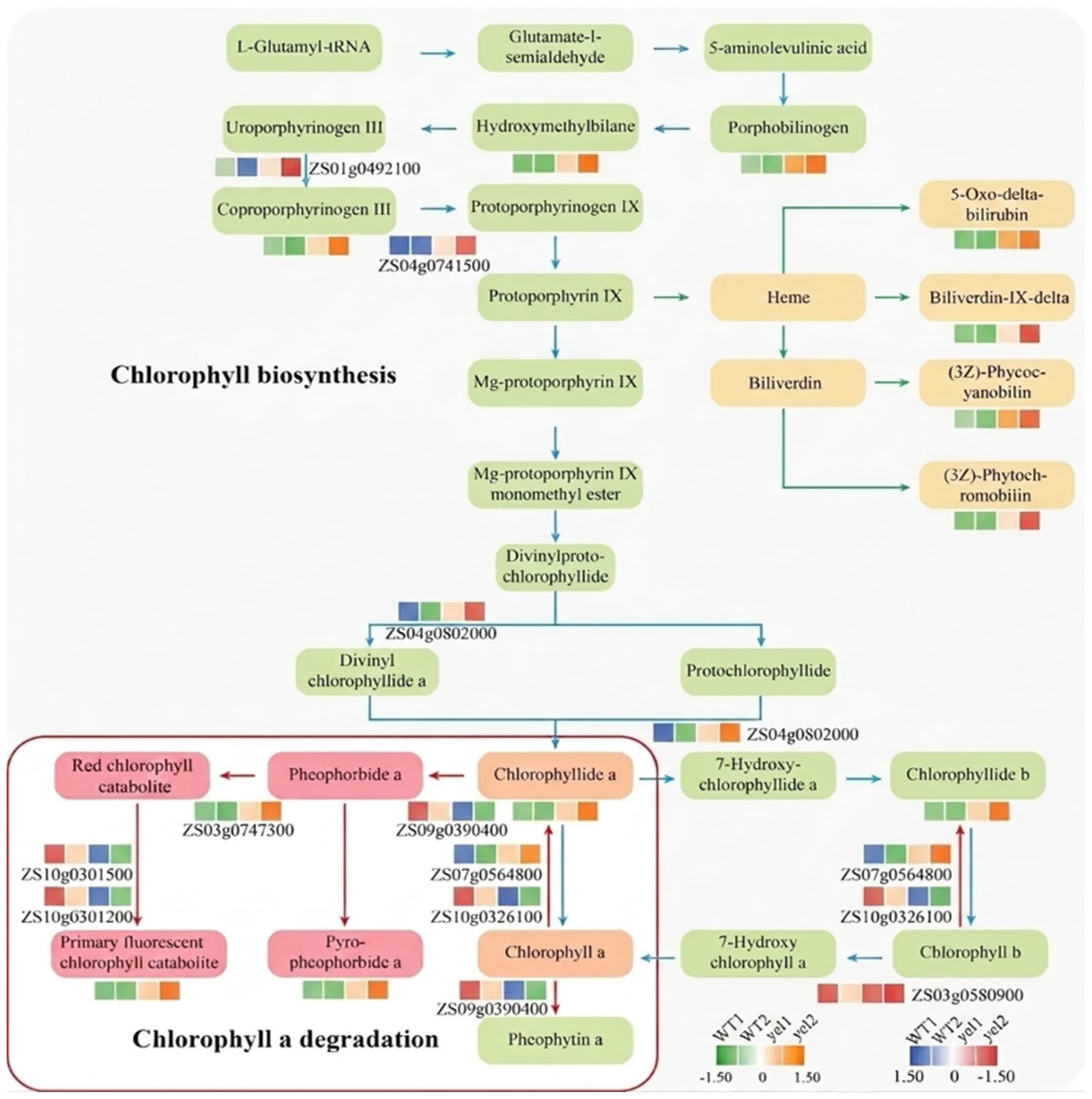
The impact of the leaf color mutation on the chlorophyll biosynthesis and metabolism pathways in the yellow−green leaf mutant.

Integrated transcriptomic and metabolomic analysis revealed a dysfunctional chlorophyll metabolic network in the *yel* mutant (Figure 10). This network exhibited contradictory reprogramming. In the synthesis pathway, several upstream genes were upregulated, leading to precursor accumulation. Conversely, a key downstream gene, *OsPORA* (Os04g0802000), was markedly suppressed. This created an upstream push, downstream bottleneck dynamic. As a result, chlorophyll precursors and chlorophylls a/b paradoxically over-accumulated during the yel1 and yel2 stages. This was not a mark of efficient synthesis, but a non-functional buildup of metabolites. Concurrently, the degradation pathway was transcriptionally activated, with key genes such as chlorophyllase (*OsCHL*) and red chlorophyll catabolite reductase (*OsRCCR*) being significantly upregulated. This activation corresponded with an increased accumulation of chlorophyll degradation products. Thus, the yellow-leaf phenotype in the *yel* mutant is not caused by a simple production shutdown. It is the direct consequence of a complex dysregulation: a two-front failure marked by the toxic accumulation of non-functional precursors from a bottlenecked synthesis pathway and the simultaneous, systemic activation of the degradation pathway.

#### 3.10.2. Metabolic Dysregulation Triggers Systemic Hormone Signal Reprogramming

**Figure 11:**
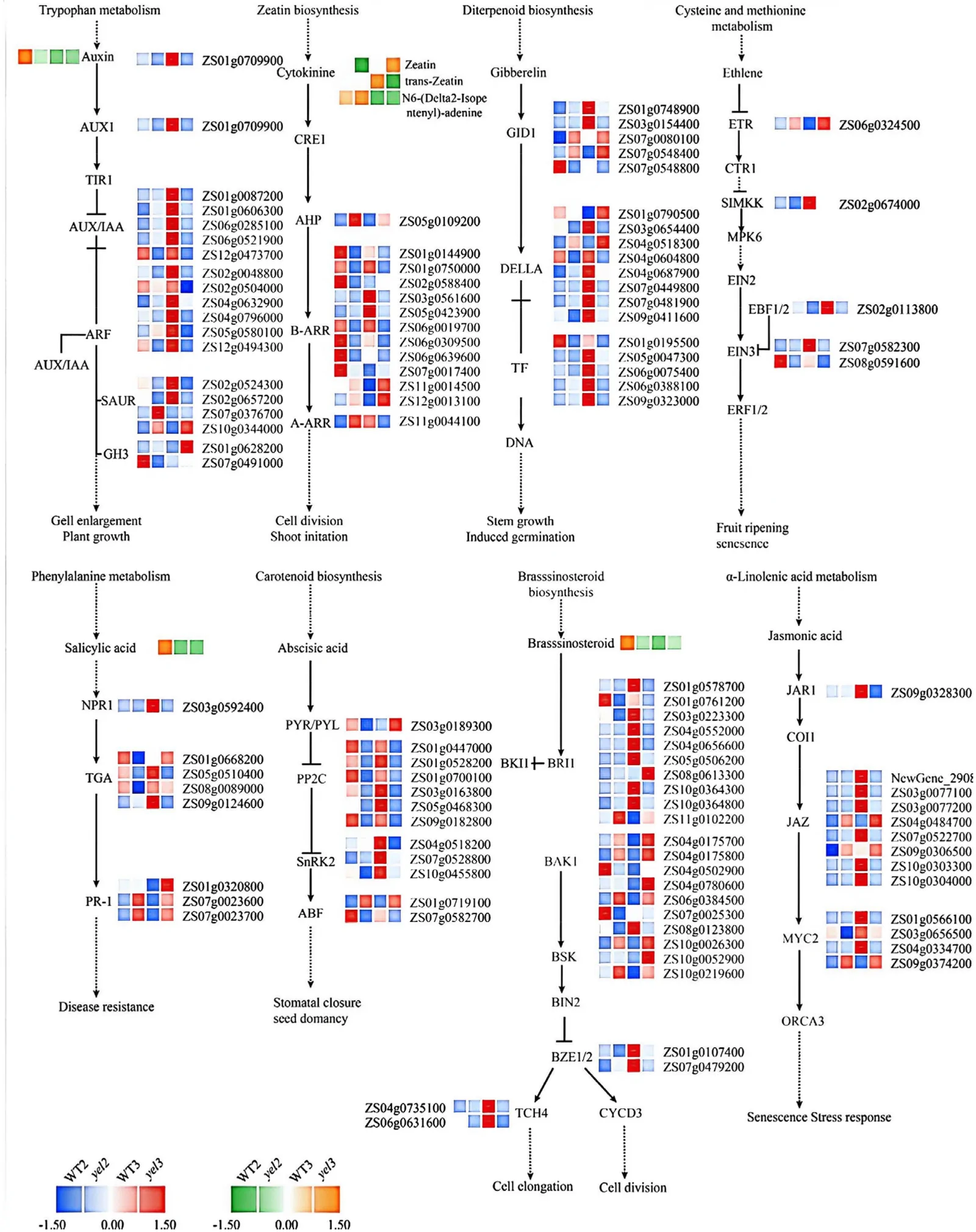
Reprogramming of plant hormone signal transduction pathways in the *yel* mutant.

The disruption of chlorophyll homeostasis triggered a systemic reprogramming of the plant hormone signaling network. This reprogramming was defined by a decisive shift from pro-growth to a stress and senescence mode (Figure 11). The suppression of growth signals was evident first. Key signaling pathways for auxin, cytokinin, and gibberellin (GA) were broadly downregulated. Critically, the master growth-repressing DELLA protein was upregulated, providing a direct molecular explanation for the mutant dwarf phenotype. In direct opposition, four major hormone signaling pathways dedicated to stress and senescence—ethylene, jasmonic acid (JA), salicylic acid (SA), and abscisic acid (ABA)—were concurrently activated. This was marked by the significant induction of key positive transcription factors, including EIN3, MYC2, NPR1, and OsABF1. The activation of these factors served as a molecular switch, launching system-wide stress-defense and leaf senescence programs.

#### 3.10.3. The Ultimate Mechanism: A Vicious Cycle Exacerbating the Phenotype

Ultimately, the integrative analysis revealed a clear causal chain forming a vicious cycle. The activation of stress hormone signals (especially ethylene and jasmonic acid) was not only a consequence of metabolic collapse but also a new driver that accelerated the chlorotic phenotype. These hormones are key signals promoting chlorophyll degradation and leaf senescence. Therefore, their activation effectively initiated the systemic process for the active degradation of chlorophyll.

## 4. Discussion

### 4.1. Putative MPEC Functional Defects and Associated Global Transcriptional Remodeling

Magnesium–protoporphyrin IX monomethyl ester cyclase (MPEC) is an essential enzyme in chlorophyll biosynthesis, catalyzing the formation of the isocyclic ring, a structure common to all chlorophylls [34]. The MPEC enzyme consists of several components, with its core being a membrane element that contains a diiron motif and a leucine zipper [35,36]. Genes encoding this membrane element are conserved across bacteria, algae, and plants and are classified into three major groups based on their need for accessory subunits [37]. Homologs have been identified in various species, including acsF, *Crd1*, *Cth1*, *Chl27*, *PNZIP*, and *NTZIP*. For example, *Crd1* in *Chlamydomonas* is activated under copper deficiency or anoxia [6]. In *Arabidopsis*, the *chl27* antisense plants (*chl27-as*) exhibit slow growth and low photosynthetic activity [7,30]. Other homologs in *Pharbitis nil* (*PNZIP*) and tobacco (*NTZIP*) are also involved in chlorophyll biosynthesis [38,39]. In rice, three MPEC mutants have been reported, including ygl8, yl-1, and m167, all of which display various yellow or virescent leaf phenotypes throughout their development [40,41,42]. In this study, Os05g0560300 was identified as the candidate causal gene associated with the *yel* mutant phenotype, which encodes the MPEC enzyme. Notably, no significant difference in the transcript abundance of *OsMPEC* was detected between wild-type and mutant seedlings. Combined with the identified missense mutation, we speculate that this nucleotide variation may disrupt the structural integrity or stability of the MPEC protein rather than altering gene transcription. Such putative protein dysfunction may lead to broad transcriptional suppression across the chlorophyll metabolic pathway. We further hypothesize that chloroplast-to-nucleus retrograde signaling might mediate this genome-wide transcriptional shift, though this signaling pathway requires direct experimental verification. To further identify the molecular basis of MPEC dysfunction in the *yel* mutant, we performed BSA-seq and sequence analysis.

BSA-seq and sequence analysis identified a G→T single-nucleotide substitution in the coding region of the *OsMPEC* gene (*Os05g0560300*), resulting in the replacement of glycine (Gly) with valine (Val) at amino acid position 461 (G461V). To assess the functional significance of this residue, we performed multiple sequence alignment of the conserved domains of MPEC homologs from three gramineous species—rice, sorghum, and maize (Figure 5). The alignment revealed that the glycine residue at position 461 is fully conserved across all three species, and this striking evolutionary conservation strongly suggests that this site plays an irreplaceable and critical role in the structural or catalytic function of MPEC. Glycine, being the only amino acid lacking a side chain, confers exceptional conformational flexibility to the polypeptide backbone, whereas valine carries a bulky isopropyl side chain. At such an evolutionarily constrained position, the substitution of glycine with valine (Gly→Val) constitutes a disruptive, non-conservative replacement, and the sudden increase in side-chain volume is likely to impair MPEC function through multiple mechanisms: (1) introducing steric hindrance within the conserved local region surrounding position 461, which may distort the backbone trajectory or interfere with the precise positioning of nearby functional groups; (2) if this residue resides within an α-helix or a protein–protein interaction interface, the branched side chain of valine could disrupt helix packing density or hinder the efficient assembly of MPEC with other cyclase subunits such as *CRD1*; and (3) the mutation may alter the conformational dynamics of the MPEC complex, thereby compromising its catalytic cycle efficiency. Notably, although the transcript level of *OsMPEC* itself did not differ significantly between the wild-type and the mutant, the substitution of the evolutionarily conserved G461 with Val most likely causes functional impairment at the protein level—a notion that is strongly corroborated by the metabolomics data, which revealed marked accumulation of magnesium–protoporphyrin IX monomethyl ester and its downstream metabolic intermediates (Figure 10), further supporting that this point mutation underlies the cyclase activity defect. Nevertheless, the precise effects of the G461V mutation on subcellular localization, in vivo protein abundance, and in vitro enzymatic activity of MPEC remain to be definitively determined, and future experiments such as Western blotting and enzyme activity assays will be required to validate these hypotheses.

### 4.2. Metabolic Dysregulation Triggers a Systemic Shift from Growth Arrest to Stress Response

The dysregulation of chlorophyll metabolism was the primary cause of the observed phenotype, and the resulting systemic physiological stress was a direct consequence. Our study clearly reveals that the hormone signaling network inside the mutant underwent a drastic reprogramming. This network shifted from a pro-growth mode to a stress and senescence mode, representing an inevitable response to the failure of a core metabolic process. In addition to retarded shoot growth, the *yel* mutant exhibited more severe suppression of root elongation (Figure 1). Without quantitative root length data, we explained this growth discrepancy via a carbon starvation mechanism. Roots are heterotrophic organs sustained exclusively by photosynthates from aerial leaves. The *OsMPEC* mutation impaired chlorophyll accumulation and reduced net photosynthetic rate by 42.28% (Figure 4), restricting carbohydrate translocation to roots. More reliant on newly fixed carbon than shoots, roots thus suffered greater growth inhibition. Meanwhile, activated ABA signaling (Figure 11) further restrained root elongation. Consistently, starch and sucrose metabolism pathways were markedly enriched among DEGs and DAMs at the three-leaf stage (Figure 7B and Figure 9B), indicating systemic carbohydrate metabolic disturbance. Future measurements of root sugar levels and tissue-specific hormones will validate this hypothesis. First, normal growth and development programs were severely disrupted. For instance, gibberellins (GAs) are essential for key developmental processes in plants [43]. In the GA pathway, we observed the significant upregulation of the gene encoding the DELLA protein, a key negative regulator. This transcriptional upregulation of DELLA genes correlates closely with the dwarf phenotype of the mutant, implying that enhanced DELLA-mediated growth repression may contribute to seedling stunting. However, protein-level quantification and genetic rescue experiments are required to confirm this regulatory relationship. Concurrently, the downstream chlorophyll biosynthesis gene *OsPORA* was suppressed. Such transcriptional dysregulation, together with auxin pathway perturbations, collectively appears to underlie the pronounced growth retardation and aberrant leaf morphology. The developmental transition from the two-leaf to the three-leaf stage provides a critical context for understanding the progressive intensification of these hormonal changes. At the two-leaf stage (yel1), the transcriptional response remained largely confined to core information processing pathways, with limited and statistically marginal metabolic enrichment (Figure 7A). By the three-leaf stage (yel2), however, the plant transitions to full autotrophy, imposing a sharp increase in photosynthetic demand. The compromised chlorophyll biosynthesis capacity, coupled with the accumulation of toxic porphyrin intermediates, progressively overwhelms cellular buffering mechanisms. This metabolic overload triggers a systemic stress response that manifests as the coordinated activation of ABA, JA, ethylene, and SA signaling pathways (Figure 11). Thus, the progressive intensification of leaf chlorosis and growth inhibition in the *yel* mutant is not merely a direct consequence of the primary MPEC defect, but reflects a stage-dependent collapse of metabolic homeostasis, as the plant’s energy needs outpace its compromised biosynthetic capacity. While some studies suggest a positive regulatory role for DELLA in chlorophyll biosynthesis under specific conditions [44], our data indicate that in the *yel* mutant, its canonical growth-repressing function predominates, and the chlorosis is overwhelmingly caused by the primary MPEC defect. Conversely, stress and senescence-related signaling pathways were co-activated, which in turn exacerbated the yellow-green leaf phenotype. In the abscisic acid (ABA) pathway, for example, mutations in synthesis-related genes can directly inhibit ABA production, leading to reduced ABA levels, affecting growth, and causing leaf color changes [45]. In the mutant, we observed that *OsABF1* and its downstream target, *OsSGR1*, were simultaneously upregulated [46]. According to recent studies, ABFs can directly activate *SGR1* to mediate ABA-induced chlorophyll degradation [47]. Collectively, these results lead us to hypothesize that activated ABA signaling may accelerate chlorophyll breakdown via the *OsABF1-OsSGR1* cascade. Elevated transcript levels of chlorophyll catabolic genes and accumulated degradation products support this trend, while chlorophyll degradation rate and catabolic enzyme activity measurements are needed to validate this synergistic inhibitory loop [48]. Integrating the transcriptomic and metabolomic layers, a coherent picture emerges: the transcriptional suppression of chlorophyll biosynthesis genes is consistently accompanied by the accumulation of porphyrin intermediates, whereas the coordinated upregulation of degradation genes correlates with increased levels of their corresponding catabolic products. The concordance between these two omics datasets—particularly at the level of specific pathway nodes such as MgPEC, POR, and the ABA-responsive degradation module—provides cross-layer validation that the MPEC defect drives a cascade of systemic metabolic reprogramming. Rather than relying on any single omics layer, it is this consistency across transcriptional and metabolic readouts that most strongly supports the causal chain proposed in our integrative model.

### 4.3. An Integrated Model to Explain the yel Phenotype

In summary, we put forward a plausible multi-layered molecular framework to interpret the progressive physiological and morphological phenotypes observed in the *yel* mutant. The cascade is initiated by the Gly461Val missense mutation in *OsMPEC*, which is predicted to impair MPEC protein structure and catalytic function. This putative cyclase deficiency may trigger genome-wide transcriptional repression of chlorophyll biosynthetic genes potentially through chloroplast retrograde signaling, blocking normal chlorophyll production.

This primary defect in pigment metabolism initiates widespread physiological stress across seedlings, rewires endogenous hormone signaling from growth-promoting programs toward stress adaptation and senescence responses, and diminishes photosynthetic carbon fixation efficiency. Disrupted carbohydrate distribution consequently leads to more severe growth suppression in heterotrophic roots compared with aerial tissues. Meanwhile, elevated ABA signaling is hypothesized to accelerate chlorophyll breakdown via the *OsABF1-OsSGR1* regulatory module. The combined effects of attenuated chlorophyll synthesis and ABA-triggered pigment degradation gradually result in persistent yellow-green leaf chlorosis and progressive developmental retardation. Notably, this mechanistic model is established mainly based on phenotypic observation, BSA-seq mapping, transcriptomic and metabolomic correlative data. Several critical verifications remain to be fulfilled in future work: Transgenic complementation and CRISPR knockout assays to conclusively validate the causal relationship between the *OsMPEC* mutation and the *yel* phenotype; Western blotting, enzyme activity and protein stability assays to characterize how the Gly461Val substitution alters MPEC function biochemically; Detection of canonical retrograde signaling markers and pharmacological treatments to confirm the involvement of plastid-to-nucleus signaling; DELLA protein quantification and growth rescue tests to verify its role in dwarfism formation; Direct measurement of chlorophyll degradation rates to validate the synergistic inhibitory loop of synthesis repression and catabolism acceleration. All these complementary experiments will help solidify the causal connections within the regulatory network proposed in the present study.

## 5. Conclusions

This study employed a multi-omics approach to unveil the molecular mechanism underlying the *yel* phenotype in rice. We identified the candidate causal gene as *Os05g0560300*, which encodes a magnesium–protoporphyrin IX monomethyl ester cyclase (*OsMPEC*). Although the gene transcript level was unchanged, a functional defect in the protein triggered a global transcriptional repression of the chlorophyll metabolic network.

This primary defect led to differentiated metabolic consequences. At the two-leaf stage, it resulted in blocked chlorophyll biosynthesis. At the three-leaf stage, however, it progressed to a non-functional accumulation of metabolites due to a stalled degradation pathway. At a deeper level, this metabolic dysregulation caused a systemic reprogramming of the plant hormone signaling network, shifting from a normal pro-growth mode to a stress and senescence mode. Specifically, the activation of senescence-related hormone signals, such as ethylene and jasmonic acid, accelerated chlorophyll degradation.

In summary, the phenotype of the *yel* mutant is a synergistic effect, driven by both passive synthesis inhibition and active degradation activation. These findings not only elucidate the critical role of *OsMPEC* in maintaining chlorophyll homeostasis and normal plant development but also offer profound insights and a valuable genetic resource for understanding the complex interplay between photosynthesis and whole-plant growth, as well as the molecular basis for chlorosis and yellow-green leaf phenotype in rice.

## Figures and Tables

**Table 1:** Genetic analysis of the yel mutant in F_2_ populations.

Cross	Total F_2_	Normal	Yellow	Observed	Expected	χ^2^
*yel* × Nip	611	471	140	3.36:1	3:1	1.31
Nip × *yel*	598	467	131	3.56:1	3:1	2.89
*yel* × 02428	810	623	187	3.33:1	3:1	1.48
02428 × *yel*	358	273	85	3.21:1	3:1	0.24

**Table 2:** Statistics of SNP and Indel-related region analysis.

Chromosome ID	Start	End	Size (Mb)
Chr02	4,300,320	5,059,457	0.76
Chr05	17,836,161	21,378,705	3.54
Chr05	22,149,199	22,170,268	0.02
Chr05	22,173,628	22,198,608	0.02
Total	–	–	4.34

**Table 3:** Chlorophyll metabolism genes in candidate regions.

Pathway	KoID	Variant Gene	Gene	Variant Gene All	Gene All	Gene ID	K Num
Porphyrin and chlorophyll metabolism	ko00860	2	59	268	13,871	*Os05g0270100* Os05g0336900	K01772 K04035

**Table 4:** Key differentially expresses genes in chlorophyll metabolism in the *yel* mutant.

Gene ID	Gene Name	Function	Log2FC	FDR
Os02g0027800	*OsGluRS*	Glutamyl-tRNA synthetase	+1.43~+2.41	<0.01
Os04g0802000	*OsPORA*	Protochlorophyllide reductase	−2.03~−2.48	<0.01
Os07g0564800	*OsCHL*	Chlorophyllase	−1.76~−3.01	<0.05
Os09g0390400	——	Magnesium chelatase	+1.24~+1.47	<0.05
Os10g0301200	*OsRCCR*	Red chlorophyll catabolite reductase	+2.07~+2.32	<0.01
Os10g0326100	——	Chlorophyllase	+1.21~+2.29	<0.05

**Note:** The range of log_2_FC represents observed values from two comparison groups (WT1 vs. yel1, WT2 vs. yel2). “+” indicates upregulation, while “−” indicates downregulation. “—” means no specific gene name has been assigned.

## Data Availability

The original contributions presented in this study are included in the article and Supplementary Materials. Further inquiries can be directed to the corresponding author.

## Supplementary Materials

The following supporting information can be downloaded at: https://www.mdpi.com/article/10.3390/cimb48080848/s1.
