# Supplementary material for: Integrative Multi-Omics Analysis Reveals Systemic Transcriptional and Hormonal Reprogramming Associated with a Rice Yellow-Green Leaf Mutant
Source: Curr Issues Mol Biol. 2026 Aug 20;48(8):848. doi: 10.3390/cimb48080848 (PMC13511092; doi:10.3390/cimb48080848)
Supplement: Supplementary file 1 [file cimb-48-00848-s001.zip › cimb-4474815-supplementary.pdf]

Supplementary

S1. Summary of BSA-seq data output and quality

Sample	Clean Reads	Clean Bases (Gbp)	Q20 (%)	Q30 (%)	GC Content (%)
FY	86973636	12.98	99.16	94.27	40.88
H1-G	87074790	12.98	99.15	94.23	40.97
QB	91287726	13.63	99.06	93.69	40.73
Yellow	105471820	15.72	98.56	92.29	41.55

BSA Sequencing Quality Assessment: The yellow-green leaf mutant *yel* yielded a total of 55.31 Gbp of clean data, with a Q30 of 93.62%. The average mapping rate between the sample and the reference genome was 97.95%, the average sequencing depth was 34.50×, and the genome coverage was 96.93%.(S1)

S2. Statistics of sequencing alignment and genome coverage

Sample	Mapped Reads (%)	Properly Mapped (%)	Average Depth (×)	Coverage 1×(%)	Coverage 5×(%)	Coverage 10×(%)
FY	97.02	90.27	31	94.77	91.23	87.57
H1-G	98.41	90.76	32	98.49	94.33	89.94
QB	98.43	90.24	33	91.51	86.25	82.58
Yellow	97.06	92.16	38	97.19	94.26	91.2

The parental lines FY and QB had an average genome sequencing depth of approximately 32×, with 1× genome coverage of approximately 93.14%, 5× gene coverage of 88.74%, and 10× gene coverage of 85.08%. The two bulked pools had an average sequencing depth of approximately

35×, with 1× genome coverage of approximately 97.84%, 5× gene coverage of 94.29%, and 10× gene coverage of 90.57%.(S2)

S3 Statistical Table of SNP-associated Region Analysis

**Chromosome ID**	**Start**	**End**	**Size (Mb)**
Chr02	3,634,342	3,634,358	0
Chr02	3,635,127	3,818,302	0.18
Chr02	3,818,977	3,820,021	0
Chr02	3,823,473	3,823,473	0
Chr02	3,824,342	3,825,314	0
Chr02	3,890,398	4,128,143	0.24
Chr02	4,300,320	5,121,570	0.82
Chr02	5,123,008	5,125,988	0
Chr05	17,834,785	17,834,835	0
Chr05	17,836,161	21,378,705	3.54
Chr05	22,116,217	22,116,351	0
Chr05	22,117,835	22,117,835	0
Chr05	22,118,197	22,227,706	0.11
Total	—	—	4.89

Prior to the association analysis, a series of stringent filtering steps were first applied to the SNPs. Specifically, SNP loci with multiple genotypes were excluded, and only biallelic loci were retained. Second, SNP loci with a pooled read support of less than 4 were removed. Third, SNP loci that were homozygous and identical between the two bulked pools, SNP

loci that were homozygous and identical between the two parental lines, and SNP loci where the recessive bulk genotype was not derived from the recessive parent were eliminated. Finally, SNP loci where the dominant bulk genotype was not derived from the dominant parent were excluded. After these filtering steps, a total of 1,439,094 high-quality and reliable SNP loci were obtained.

This study employed two association analysis methods to investigate genomic regions associated with the trait. First, the SNP-index association algorithm was used for association analysis. At a confidence level of 99, a total of 37 regions with a combined length of 16.49 Mb were identified. Next, the ED association algorithm was applied, using the median of the fitted values for all loci plus three times the standard deviation as the association threshold, yielding a threshold value of 0.47. Based on this threshold, a total of 13 regions with a combined length of 4.90 Mb were obtained. Finally, the SNP-associated regions identified by the two association analysis methods were intersected, revealing 13 candidate regions on chromosomes Chr02 and Chr05, with a total length of 4.89 Mb.(S3)

S4 Statistics of Indel-related region analysis

Chromosome ID	Start	End	Size (Mb)
Chr02	4,188,960	5,059,457	0.87
Chr05	17,835,672	22,107,707	4.27
Chr05	22,117,058	22,117,119	0
Chr05	22,149,199	22,170,268	0.02
Chr05	22,173,628	22,198,608	0.02
Total	–	–	5.18

First, InDels were filtered using the same criteria as those applied in the SNP analysis, yielding a total of 290,430 high-quality and reliable InDel loci. During the association analysis stage, the median + 3SD of the fitted values for all loci was used to determine an association threshold of 0.45. Based on this threshold, a total of 5 regions with a combined length of 5.19 Mb were identified. At a confidence level of 99, 2 regions with a total length of 14.13 Mb were obtained. The intersection of the InDel-associated regions identified by the two association analysis methods yielded a total of 5 regions with a combined length of 5.18 Mb.(S4)

S5 Statistics of gene functional annotation within candidate regions

Annotated Databases	Gene Num	Non-Syn Gene Num	Frame Shift Gene Num
NR	604	335	94
trEMBL	640	351	102
SwissProt	283	169	49
GO	391	225	69
KEGG	321	190	60
COG	142	93	30
Total	640	351	102

The coding genes within the candidate intervals were deeply annotated using multiple databases with BLAST software to rapidly screen for candidate genes. A total of 640 genes were annotated within the candidate regions, among which 351 non-synonymous mutation genes and 102 frameshift mutation genes were identified between the parental lines. Specifically, there were 19 genes on chromosome Chr02 and 287 genes on chromosome Chr05.(S5)

S6 GO screening of chlorophyll metabolism-related genes within candidate regions

Go Term	GO ID	Variant Gene	Gene	Variant Gene All	Gene All
Biological Process: chlorophyll metabolic process (GO:0015994)	2	5	191	16540	OsZS05G0323900<br>OsZS05G0323800

Biological Process: chloroplast mRNA processing (GO:0010239)	1	7	191	16540	OsZS02G0067600
Cellular Component: chloroplast thylakoid (GO:0009534)	2	49	133	14164	OsZS05G0323800<br>OsZS05G0323900
Cellular Component: chloroplast (GO:0009507)	4	500	133	14164	OsZS02G0067600<br>OsZS02G0075300<br>OsZS05G0334900
Cellular Component: chloroplast envelope (GO:0009941)	3	133	133	14164	OsZS05G0309800<br>OsZS05G0323800<br>OsZS05G0323900<br>OsZS02G0080100
Cellular Component: chloroplast stroma (GO:0009570)	2	186	133	14164	OsZS02G0080100<br>OsZS02G0071600
Cellular Component: chloroplast thylakoid membrane (GO:0009535)	1	132	133	14164	OsZS05G0310000
Cellular Component: chloroplast outer membrane (GO:0009707)	1	24	133	14164	OsZS05G0300600
Molecular Function:					OsZS05G0323800

chlorophyllase activity (GO:0047746)	1	2	264	26930

GO enrichment analysis of the genes within the candidate regions revealed that 10 genes associated with chloroplast development or chlorophyll synthesis and metabolism were enriched in biological processes such as chlorophyll metabolic process (GO:0015994) and chloroplast mRNA processing (GO:0010239); cellular components including chloroplast thylakoid (GO:0009534), chloroplast (GO:0009507), chloroplast outer envelope (GO:0009941), chloroplast stroma (GO:0009570), chloroplast thylakoid membrane (GO:0009535), and chloroplast outer membrane (GO:0009707); as well as the molecular function of chlorophyllase activity (GO:0047746).(S6)
